# An Efficient Approach Using Knowledge Distillation Methods to Stabilize Performance in a Lightweight Top-Down Posture Estimation Network

**DOI:** 10.3390/s21227640

**Published:** 2021-11-17

**Authors:** Changhyun Park, Hean Sung Lee, Woo Jin Kim, Han Byeol Bae, Jaeho Lee, Sangyoun Lee

**Affiliations:** 1 Department of Electrical and Electronic Engineering, Yonsei University, Seoul 03722, Korea; qkrckd2002@yonsei.ac.kr (C.P.); hslee2860@yonsei.ac.kr (H.S.L.); woojinkim0207@yonsei.ac.kr (W.J.K.); jhlee430@yonsei.ac.kr (J.L.); 2 Department of Artificial Intelligence Convergence, Kwangju Women’s University, Gwangju 62396, Korea; kwu_BHB@kwu.ac.kr

**Keywords:** pose estimation, convolutional neural network, lightweight, knowledge distillation

## Abstract

Multi-person pose estimation has been gaining considerable interest due to its use in several real-world applications, such as activity recognition, motion capture, and augmented reality. Although the improvement of the accuracy and speed of multi-person pose estimation techniques has been recently studied, limitations still exist in balancing these two aspects. In this paper, a novel knowledge distilled lightweight top-down pose network (KDLPN) is proposed that balances computational complexity and accuracy. For the first time in multi-person pose estimation, a network that reduces computational complexity by applying a “Pelee” structure and shuffles pixels in the dense upsampling convolution layer to reduce the number of channels is presented. Furthermore, to prevent performance degradation because of the reduced computational complexity, knowledge distillation is applied to establish the pose estimation network as a teacher network. The method performance is evaluated on the MSCOCO dataset. Experimental results demonstrate that our KDLPN network significantly reduces 95% of the parameters required by state-of-the-art methods with minimal performance degradation. Moreover, our method is compared with other pose estimation methods to substantiate the importance of computational complexity reduction and its effectiveness.

## 1. Introduction

The demand for human pose estimation has increased over time as it is essential for detecting human behaviors and for numerous applications such as human-computer interaction [1], human action recognition [2], and human performance analysis [3]. Previously, human pose estimation has been studied as a close-up technique requiring a balance between accuracy and low computational complexity. Traditional approaches such as histogram of oriented gradient (HOG) [4] and Edgelet [5] extract discriminative features from images and assign a class to the feature vector. However, they cannot adequately determine the accurate location of body parts in a human figure [6].

Recent advances in convolutional neural networks (CNNs) that enable robust feature extraction have afforded significant improvements in pose estimation. Therefore, owing to the feature extraction capabilities of CNNs, the research paradigm of human pose estimation shifted from classic approaches to deep learning [7,8,9]. Two main approaches, i.e., bottom-up and top-down approaches, of deep-learning-based methods, have been employed to overcome the limitations of handcrafting-based methods during the transition.

Bottom-up approaches [10,11,12,13,14,15,16] first detect human body poses and then group them using clustering algorithms. Compared to top-down approaches, they are faster in testing and thus require lower computational complexities during model building. However, the bottom-up approaches are unable to amplify the details of each person, and subsequently, they yield lower accuracies than top-down approaches.

In contrast, the keypoint prediction process in top-down approaches is a two-step operation. Generally, top-down approaches [17,18,19,20,21,22,23] first detect all the people in an image and crop the person region and then input the cropped image into a single-person pose estimation model. Due to the two-step operation, they yield better results than bottom-up approaches. To accurately estimate the keypoints of people in an image, top-down approaches construct network layers deeper than bottom-up approaches. However, top-down approaches are unable to solve the speed degradation issue that arises when deeply constructing network layers for estimating keypoints.

Most previous multi-person estimation methods require high computational complexity to accurately estimate the keypoints of people in an image. Additionally, to guarantee accuracy, the network layers need to be deeply designed, which decreases the estimation speed. Due to these limitations, the accuracy and speed need to be balanced in multi-person estimations.

In this paper, we present a lightweight top-down human pose estimation network that uses a knowledge distillation method to overcome the balance limitations. Inspired by PeleeNet [24], we propose knowledge distilled lightweight top-down pose network (KDLPN), a network that minimizes computational complexity while shuffling the pixels in the decoder previously introduced in the dense upsampling convolution (DUC) layer [25] to reduce the number of channels.

To effectively and efficiently resolve the performance degradation occurring in the lightweight networks, the knowledge distillation approach [26] is applied in our model. To satisfy the complexity and performance requirements of deploying state-of-the-art deep neural models in our proposed system, compact and fast models are trained by transferring knowledge from extremely deep and powerful teacher models. Additionally, experiments are conducted on the MSCOCO dataset [27], a widely-used human pose estimation dataset to verify the model effectiveness, and the balance between the estimation accuracy and the speed of our approach is demonstrated.

The remainder of this paper is organized as follows. Section 2 presents a review of the related work. In Section 3, the proposed two-dimensional (2D) human pose estimation model is presented and the features of each part are formulated. Section 3.1 and Section 3.2 discuss the study motivations. Section 3.3 describes the building of a lightweight 2D human pose estimation network and replacement of the decoder to reduce complexity. In Section 3.4, to prevent performance degradation, the proposed method is adopted for knowledge distillation in training. Section 4 presents the simulation results and discussion on the MSCOCO dataset. Finally, Section 4.5 presents the conclusions.

## 2. Related Work

### 2.1. Multi-Person Pose Estimation

Recently, multi-person pose estimation has drawn increasing attention because of its applicability in real-life applications, such as postural correction [28], action recognition [29], and health care [30]. Multi-person pose estimation using neural networks can be determined via two main approaches. The first is the bottom-up approaches that obtain all the pose keypoints in input images and assemble them as distinct people using methods such as part affinity field (PAF) [11]. The other is the top-down approaches that employ human detection to obtain bounding boxes and input the cropped image batch to the pose estimation neural network.

**Bottom-up approach:** Bottom-up pose estimation methods [10,11,12,13,14,15,16] detect the identity-free human joints of all people within an image and assemble the joints using an algorithm. While traditional multi-person pose estimation models focus on human structural characteristics, contemporary models focus more on measuring the body itself by adopting strong CNN models.

**Figure 1:**
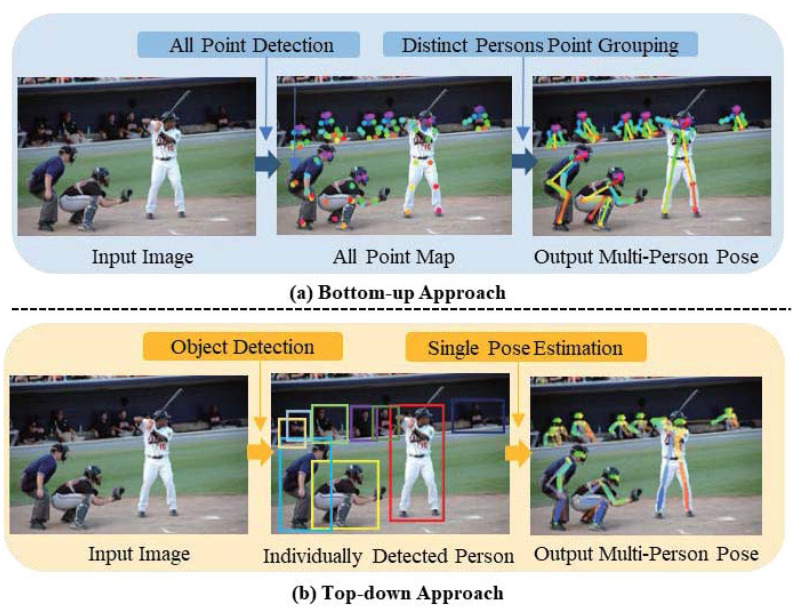
Human Pose Estimation algorithm. Column (**a**): The example of a bottom-up approach. Column (**b**): The example of a top-down approach.

To accurately estimate multi-person poses, DeepCut [12] proposed the derivation of a joint detection and pose estimation formulation that was casted as an integer linear program problem and a new formulation that was casted as a joint subset partitioning and labeling problem. Further, DeeperCut [13], an improved DeepCut employs a residual network (ResNet) [31] to extract more robust body parts representations. Moreover, it adopts image-conditioned pairwise terms to achieve better performance. Next, Associative Embedding [14] detects heatmaps with pixelwise embedding and groups the keypoint candidates by comparing the distance between the embeddings to generate the result. To improve the estimation accuracy, Openpose [11] employed PAFs to learn to associate body parts with individuals in an image. Openpose obtains a heatmap around the grouped joints of people using a multi-stage CNN (initialized by the first ten layers of VGG-19 [32]) and fine-tunes and then yields multi-person poses using PAFs (Figure 1a).

Current bottom-up pose estimation approaches have high estimation speeds, and can be implemented in mobile devices without additional human detection networks. However, their performances are significantly affected by complex backgrounds and human outer walls.

**Top-down approach:** Top-down approaches [17,18,19,20,21,22,23] employ a two-step process to estimate pose keypoints. They first detect all the people within an image using object detection. Then, each cropped image is processed into a single-person pose estimation network model. A Cascaded Pyramid Network (CPN) [20] has been proposed to robustly detect “hard” keypoints and divide keypoints into simple levels. CPN comprises a pyramid architecture as the backbone network, including GlobalNet and RefineNet. In RefineNet, CPN selects the hard keypoints online based on the training L2 loss. George et al. [19] predicted heatmaps and offsets using a fully convolutional ResNet and a faster RCNN detector to detect bounding boxes, and they then predicted the final location output using the heatmaps, offsets, and keypoint-based non maximum suppression. Regional multi-person pose estimation (RMPE) [17] comprises a human detection model and a skeleton registration model [33,34,35,36,37] for estimating the multi-person poses in the image. The detected single human bounding boxes in batches from the detection model are input into the skeleton registration model to detect the skeleton keypoints (Figure 1b).

To utilize the characteristics of the superior performance of top-down approaches and overcome its shortcomings, we propose a lightweight top-down pose estimation approach to improve computational efficiency while improving the performance.

### 2.2. Lightweight Neural Network

The effectiveness of neural networks has significantly improved the performance of applications that use various memory locations and operations. However, computing power and memory have not kept up with the development of neural networks. Consequently, lightweight networks with low computational complexity have been proposed to meet the demand for mobile devices.

MobileNets [38,39,40] proposed the construction of a lightweight model that can run on mobile devices by minimizing the number of network parameters. It minimizes the overall computation using a depth-wise convolution to convert each channel into its respective kernel and by applying a 1×1 convolution to change the output channel to pointwise convolution. MobileNetsV3 [40] proposed the platform-aware network architecture search method, which automatically optimizes each network block. It utilizes a module based on squeeze and excitation in the bottleneck structure to minimize the network parameters while improving the performance. PeleeNet [24] is a network model that performs various tunings based on DenseNet [41] for mobile devices. It utilizes the architecture of DenseNet that concatenates the feature map of the layers. Additionally, it uses stemblock and two-way dense layers to reduce the computational cost and adopts a structure with varying number of layers on each stage. The computational complexity of PeleeNet is significantly low, which allows its operation in mobile devices. It affords better accuracy and over 1.8 times faster speed than MobileNet and MobileNetV2 on the ImageNet ILSVRC 2012 dataset [42].

### 2.3. Knowledge Distillation

Deep learning models are basically wide and deep; thus, feature extension works efficiently if the number of parameters and operations are high. Subsequently, the object classification or detection performance, which is the purpose of the model, is improved. However, deep learning cannot be configured using large and deep networks owing to device limitations, such as computing resources (CPU and GPU) and memory. Therefore, considering these device environments, a deep learning model with a small size and improved performance is required. This demand has led to the development of various algorithms that can afford similar performance to large networks, and among them, knowledge distillation is attracting immense attention [26,43].

Knowledge distillation is the information transfer between different neural networks with distinct capacities. Bucilua et al. [44] were the first to propose model compression to use the information from a large model for the training of a small model without a substantial drop in accuracy. This is mainly based on the idea that student models reflect teacher models and afford similar performances. Hinton et al. [43] employed a well-trained large and complex network to help train a small network. Yim et al. [45] compared an original network and a network trained using the original network, as a teacher network. They determined that the student network that learned the distilled knowledge is optimized much quicker than the original model, and it outperforms the original network.

This is because the teacher model provides extra supervision in the form of class probabilities, feature representations [46,47], or an inter-layer flow. Recently, this principle has also been applied to accelerate the model training process of large-scale distributed neural networks and transfer knowledge between multiple layers [48] or between multiple training states [49]. In addition to the conventional two-stage training-based offline distillation, one-stage online knowledge distillation has been attempted, and advantageously, it provides more efficient optimization and learning. Furthermore, knowledge distillation has been used to distil easy-to-train large networks into harder-to-train small networks. Alashkar et al. [50] presented a makeup recommendation and synthesis system wherein the makeup art domain knowledge and makeup expert experience are both incorporated into a neural network to boost the performance of the makeup recommendation. Although knowledge distillation in deep neural networks has been successfully applied to solve the problems of visual relationship detection, sentence sentiment analysis and name entity recognition, its application in the fashion domain has been limited.

In this work, we adopt a knowledge distillation method to advantageously employ the complex teacher network knowledge to guide lightweight neural models.

## 3. Proposed Method

### 3.1. Overview

In this section, we propose a lightweight multi-person pose estimation network using a top-down-based approach. The top-down method basically comprises a detector, which detects people, and a single-person pose estimation (SPPE), which predicts a single pose from the detected person. Although the speed reduces based on the number of people in the top-down approach compared to the bottom-up approach, the top-down approach affords better performance. Moreover, the speed problem can be alleviated by minimizing the number of network parameters. As several fast and accurate approaches [33,34,35,36,37] exist for human detection, we mainly focus on making the SPPE of the pose estimation model lightweight.

**Figure 2:**
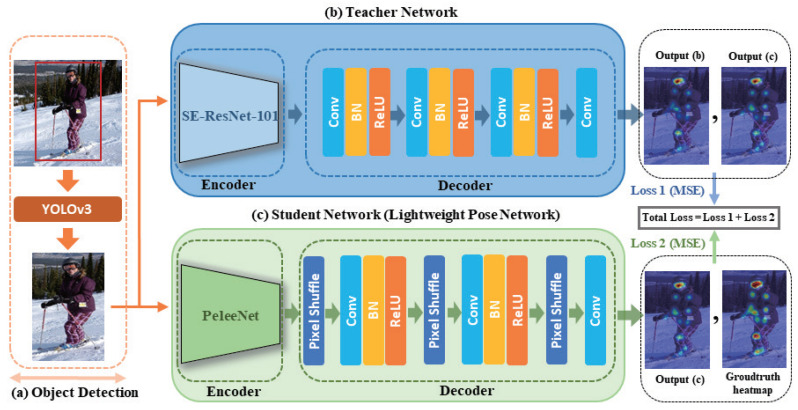
Overall lightweight human pose estimation network.

SPPE comprises an encoder model, which extracts features from the detected person as input, and a decoder model, which acquires the heatmap to the keypoints of that person by upsampling from the extracted features. As shown in Figure 2, we changed the encoder model to the proposed optimal lightweight model. Concurrently, we reduced the number of parameters by applying a new structure to the upsampling layer of the decoder model. To avoid the performance degradation when reducing the number of parameters, we employed knowledge distillation using a teacher network with high performance.

In the next section, we present the overview of our method. Then, we illustrate the lightweight network corresponding to the top-down-based SPPE in Section 3.2 and the decoder of the lightweight network in Section 3.3. Finally, we present the knowledge distillation method that can minimize the performance reduction associated with lightweightedness in Section 3.4.

### 3.2. Preliminary Processing

Human pose estimation aims to localize the body joints of all the detected people in a given image. In the top-down mode, the detector first yields the bounding box of detection information about people in images.

We use YOLOV3 [37] to quickly and efficiently detect people. The detected images are passed through a spatial transformer network [51], which is a parametric network that automatically selects areas of interest and appears prior to the SPPE input, and the detected information about the human region is converted into high quality information of the same size. Then, using the converted detection information, the SPPE extracts the heatmap, which represents the location information of the human body joints. The original resolution and size of the extracted heatmap (H) is determined by the inverse conversion of the spatial de-transformer network. Finally, we estimate the posture of every person in the image by connecting the body joints based on the heat maps extracted from each person.

### 3.3. Network Architecture

#### 3.3.1. Lightweight Network Encoder

Top-down methods, which detect people from images and estimate poses from within bounding boxes, are more accurate than bottom-up methods, which estimate all the keypoints in an image and correlate them. However, disadvantageously, in top-down methods, the detected bounding boxes need to be cropped and the estimation speed reduces if multiple people are present in the images.

Although many studies have been conducted on top-down methods [17,18,19,20,21,22,23], the limitations of heavy and slow models have not yet been overcome. As a representative example, Alpha-pose based on RMPE [17,18] utilizes a very heavy encoder structure with SE-ResNet. Therefore, after conducting multiple experiments to determine a suitable encoder structure that lightens the multi-person pose estimation network, we selected PeleeNet as the optimal encoder structure. PeleeNet is a lightweight model of DenseNet [41] and has been widely used as a feature extractor that reduces the size of the input image by four times the width and length, which makes the entire architecture cost-effective. Additionally, it can increase the feature expression ability with a small amount of computation. Moreover, to obtain the receptive fields at various scales, PeleeNet utilizes a two-way dense layer, where DenseNet only comprises a combination of 1 × 1 convolution and a 3 × 3 convolutions in the bottleneck layer. Instead of a depth-wise convolution layer, it utilizes a simple convolution layer to improve its implementation efficiency. Owing to its efficient methods and small number of calculations, its speed and performance are superior to those of typical methods, such as MobileNetV1 [38], V2 [39], and ShuffleNet [52]. Furthermore, because of its simple convolution, the use of additional techniques could likely afford a much more efficient detector. Various types of network decoders can be added via simple convolutions of the encoder while applying various training methods.

#### 3.3.2. Lightweight Network Decoder

**Figure 3:**
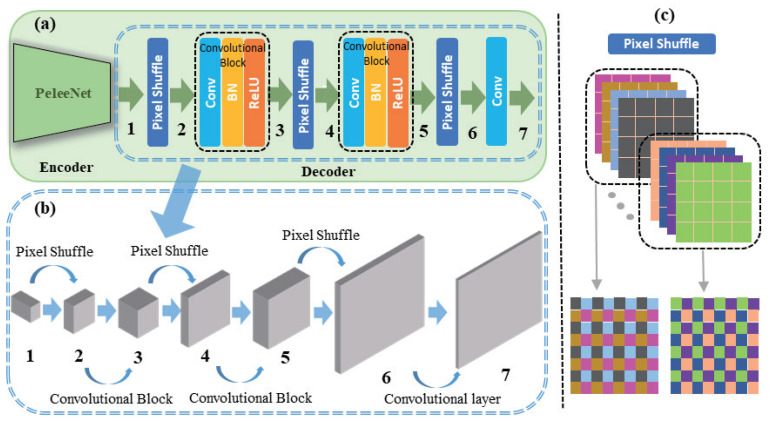
Specifications of the decoder of our proposed algorithm. (**a**): Block diagram of proposed algorithm. (**b**): The process of decoding. (**c**): The example operation of PixelShuffle.

To speed up the computation in the decoder, we designed a novel network structure using the DUC proposed in Figure 3. Table 1 summarizes the structure of the entire decoder comprising the proposed DUC layer. The DUC layer contains pixel shuffle operations, which increase the resolution and reduce the number of channels, and 3 × 3 convolution operations. When the input feature map is set to (*H*) × width (*W*) × channel (*C*), pixel shuffle reduces the number of channels to *C*/$d^{2}$ and increases the resolution to *dH* × *dW* as shown in Figure 3. Here, *d* denotes the upsampling coefficient and is set as 2, i.e., the same as that in the standard deconvolution-based upsampling method. This helps substantially reduce the number of parameters to *C*/$d^{2}$ during upsampling. The feature that reduces the channel to *C*/$d^{2}$ size using the pixel shuffle layer again expands the number of channels to *C*/d through the convolution layer. This minimizes performance degradation by embedding the same amount of information into the feature as that before the reduction of the number of input channels. The entire decoder structure includes three DUC layers and outputs heatmaps showing the positions of each keypoint in the last layer. The proposed decoder network substantially reduces the number of parameters and speeds up the computation compared to the standard deconvolution-based decoder.

### 3.4. Knowledge Distillation Method

Accuracy and speed must both be considered in multi-person pose estimation. However, most existing methods only focus on accuracy and thus consume considerable computing resources and memory. However, lightweight networks exhibit performance degradation because of the reduced computing resources.

To overcome these shortcomings, we applied knowledge distillation to alleviate the performance degradation of the lightweight multi-person pose estimation network.

(1). We trained a large pose model of the teacher network. Then, we selected SE-ResNet-101 as the teacher network because it utilizes squeeze-and-excitation blocks to perform channel-wise feature extraction.

(2). Thereafter, we trained a target student model using the knowledge learned by the teacher model. The training model is capable of handling wrong pose joint annotations, e.g., when the pretrained teacher predicts more accurate joints than the manually assigned wrong and missing labels.

As stated by [26], knowledge distillation mainly aims to designs an appropriate mimicry loss function that can effectively extract a teachers’ knowledge and transfer it to student model training. The previous distillation functions were designed for single-label based softmax cross-entropy loss in the context of object categorization and are thus unsuitable for transferring the structured pose knowledge in a 2D image space.

To address this problem, we employed a joint confidence map dedicated pose distillation loss function, as given below:

(1) $$L_{total}=\alpha_{KD}\left(\frac{1}{N}\sum_{n=0}^{N}{\left({m_{n}}^{S}-{m_{n}}^{GT}\right)}^{2}\right)+\left(1-\alpha_{KD}\right)\left(\frac{1}{N}\sum_{n=0}^{N}({m_{n}}^{S}-{{m_{n}}^{T})}^{2}\right)$$

Here, $\alpha_{KD}$ is the knowledge distillation balancing parameter. Additionally, *N* denotes the number of joints, and ${m_{n}}^{S}$, ${m_{n}}^{T}$, and ${m_{n}}^{GT}$ denote the heatmaps for the n-th joint predicted by the in-training student target model, pretrained teacher model, and corresponding ground truth of the prediction, respectively. Then, to maximize the comparability with the pose supervised learning loss, we set the mean squared error as the distillation quantity to measure the divergence between the estimation and its label. By employing these knowledge distillation techniques, learning was performed using superior and complex networks rather than the existing ground truth alone, which boosted the performance of lightweight networks to match that of the superior networks.

## 4. Experiments and Results

### 4.1. Dataset and Evaluation Matrix

We used the MSCOCO dataset [27] to train and evaluate our method. The dataset comprises more than 200 k images including 250 k person instances with 17 keypoints per instance. We trained our method on the training set of the MSCOCO dataset, comprising 56 k images including 150 k person instances. We used the official evaluation metric of the MSCOCO keypoints challenge dataset, i.e., average precision (AP) based on object keypoint similarity (OKS). OKS is a measure of how close a predicted keypoint is to the ground truth, and is defined as follows:

(2) $$OKS=\frac{\sum_{i}exp\left(-d_{i}^{2}/2s^{2}k_{i}^{2}\right)\delta \left(v_{i}>0\right)}{\sum_{i}\delta \left(v_{i}>0\right)},$$

where $d_{i}$ is the Euclidean distance between a detected keypoint and its corresponding ground truth, $v_{i}$ is the visibility flag of the ground truth, *s* is the object scale, and $k_{i}$ is a per-keypoint constant that controls fall off. We report the standard AP and recall scores from MSCOCO dataset: $AP^{50}$ (AP at OKS = 0.50), $AP^{75}$, AP (the mean of AP scores at OKS = 0.50, 0.55, …, 0.90, and 0.95), $AP^{M}$ for medium objects, $AP^{L}$ for large objects, and AR (the mean of recalls at OKS = 0.50, 0.55, …, 0.90 and 0.95).

### 4.2. Training Details

A YOLOV3 detector that was pretrained on the MSCOCO dataset was utilized to detect humans in images. Each detected image was resized to 384×256 and was randomly flipped horizontally to augment the data. PeleeNet was used as the encoder of the proposed method, and it was pretrained using ImageNet. The model was trained for 120 epochs, and the initial learning rate was set to 0.0001, which decreased by 10% at both the 60th and 90th epochs. Then, the model was optimized using an Adam optimizer [53] and the batch size was set as 8. An SE-ResNet-based RMPE that was pretrained on the MSCOCO dataset was used as the teacher network, and the knowledge distillation parameter alpha was set as 0.8.

### 4.3. Ablation Study

#### 4.3.1. Lightweight Network Structure

Additional experiments were conducted to evaluate the effectiveness of employing PeleeNet as the backbone for human pose estimation. The number of parameters (*M*) and FLOPS (*G*) and *AP* were measured using various encode models and compared PeleeNet, the encoder of our model, was compared with popular lightweight networks such as MobileNetV1, V2, and V3 [38,39,40], ShuffleNetV2 [54], MnasNet [55], and Hourglass [56] with 1, 2, and 4 stacks. The knowledge distillation parameter $\alpha_{KD}$ was set as 0.8. The results are summarized in Table 2.

**Figure 4:**
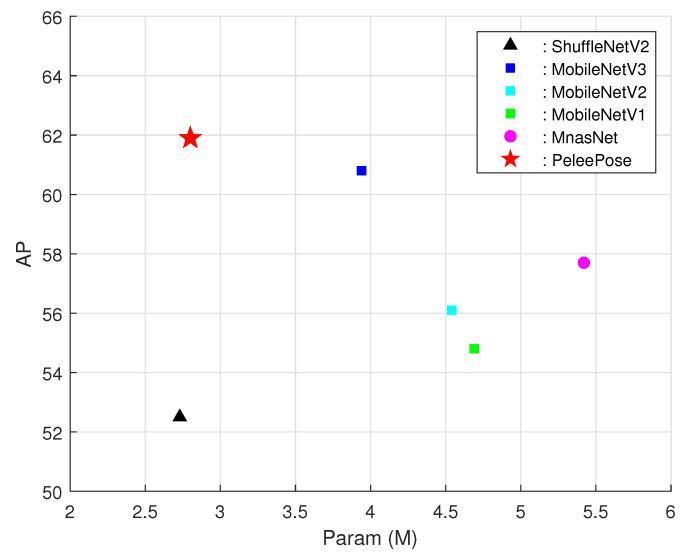
Comparison of the parameters and accuracies of lightweight networks for MSCOCO validation Sets.

As shown in Table 2, from the perspective of AP, PeleeNet affords better performance than the other encoders. Moreover, PeleeNet achieves significantly better accuracy and lower complexity than MobileNetV1, V2, and V3 and MnasNet. Compared to ShuffleNetV2, PeleeNet exhibits better AP by 7.1. Although models with Hourglass with 2 stacks and Hourglass with 4 stacks exhibited better accuracy than our KDLPN, the number of their network parameters was significantly higher. The table also shows that when PeleeNet is used as the encoder, stable performance can be obtained even with a small number of parameters. Figure 4 shows a schematic diagram of Table 2.

#### 4.3.2. Decoder Structure

To select the best decoder for KDLPN, we performed experiments to evaluate the performance of decoder approaches using the knowledge distillation method with overall loss alpha $\alpha_{KD}=0.8$. In comparison, we also attempted to implement a three-step deconvolution layer decoder. For the three-step deconvolution layer decoder, experiments were performed by changing the number of channels from 352 to 44 for each decoder layer condition. To evaluate the accuracy and efficiency, Table 3 shows comparison of the number of parameters in the networks.

In Table 3, the parameters in the proposed decoder are reduced as compared to the parameters of the deconvolution decoder. From the computational complexity perspective, KDLPN with DUC exhibits the best performance. It uses only 38% of the parameters but affords a competitive performance to the deconvolution decoder with an AP difference of 1.5. The parameter and FLOPS of this decoder model were reduced to nearly seven and ten times that of deconvolution decoder, respectively, with competitive performance.

To demonstrate the effectiveness of our method, we performed various experiments such as simplifying the number of channels, and simply reducing the parameters of the proposed DUC model, and measured the corresponding performance and complexity. First, we constructed a baseline model by combining the encoder of the PeleeNet and a decoder comprising three deconvolution layers. Then the number of deconvolution channels in the lightweight model was modified from (256, 256, 256) (baseline model) to (512, 512, 512), (128, 128, 128), (64, 64, 64) and half-step channel. The half-step channel has the same output channel size as the DUC decoder model proposed as (176, 88, 44). The resultant performance, memory size, and FLOPS obtained by lightweighting the model in the aforementioned manner are presented in Table 3. In the experiment on reducing the number of channels, highest performance was afforded when the output channel size was reduced and modified to (256, 256, 256). Models with reduced output channel size of (128, 128, 128) and (64, 64, 64) exhibited performance degradation of 1.1% and 31.8%, respectively. The performance of the proposed DUC layer reduced by 2% on average compared to the existing model; however, the FLOPS and memory size considerably reduced to 85.4% and 60.5%, respectively, compared to those of the baseline model with the output channel size of (256, 256, 256). Moreover, compared to the model with the smallest output channel size of (64, 64, 64), FLOPS and memory size decreased further to 41.0% and 17.2%, respectively. Moreover, in comparison with the half-step deconv model with the same channel standard as the DUC decoder, FLOPS and memory size decreased to 86.4% and 89.7%, respectively. This indicates that the proposed DUC method is more efficient in lightweighting than the simple reduction of the number of channels. Considering the computational cost and performance of these methods presented in Table 3, KDLPN with DUC is the optimal model that can balance accuracy and efficient performance.

#### 4.3.3. Knowledge Distillation Method

To demonstrate and optimize the effect of the knowledge distillation (Section 3.4) on the proposed network, experiments were performed on the proposed model with respect to $\alpha_{KD}$. Table 4 shows the results of the experiments with varying $\alpha_{KD}$ using the teacher network. The table also shows the APs for each overall function $\alpha_{KD}$ in the same backbone network and DUC decoder. The $\alpha_{KD}$ values were varied from 0.3 to 1.0 for each dataset. The knowledge distillation method afforded better performances across all intervals than the PeleeNet network with DUC (57.4 AP). Furthermore, $\alpha_{KD}=0.8$ afforded the best performance in this experiment; thus, we selected $\alpha_{KD}=0.8$ for model training.

**Figure 5:**
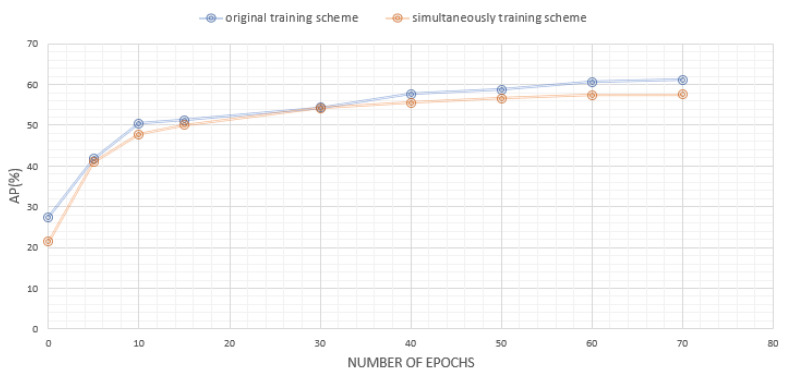
A graph comparing performance according to epoch of simultaneous training method and existing training method on MSCOCO validation dataset.

During training through knowledge distillation, the knowledge of a teacher network can be advantageously learned, which is relatively accessible compared to the ground truth, which is difficult to learn. Accordingly, we first prepared a large and deep pretrained network using the teacher network and then trained a student network to apply knowledge distillation using the teacher network. If the teacher and student networks are simultaneously trained, the performance decreases since the teacher network is not converged. Similarly, when training a teacher network that has already converged, the test performance of the student network deteriorates as the optimally trained teacher network is overfitted to the training set. Regarding the above case, we conducted additional experiments, and the graph below displays the performance comparison between the model where the teacher and student network are simultaneously trained and the original training scheme. As shown in Figure 5, the performance of the simultaneously trained model, indicated in orange color, is decreased than that of the existing model, indicated in blue color. The reason why the performance difference between the two experiments is small is that both models used the same pretrained teacher model. However, since the teacher model is already pretrained, it can be overfitted to the training set during simultaneous learning, and the performance may degrade due to the probability of deviating from the optimal point. For this reason, the proposed original training scheme shows higher performance.

### 4.4. Results and Analysis

#### 4.4.1. Overall Results

We compared our methods to other current state-of-the-art top-down-based human pose estimation methods such as RMPE, Mask-RCNN [57], and G-RMI [19]. For fair comparison, we used the same human detector for the top-down approach, to evaluate the pose estimation network performance of these methods based on a uniform criterion.

**Figure 6:**
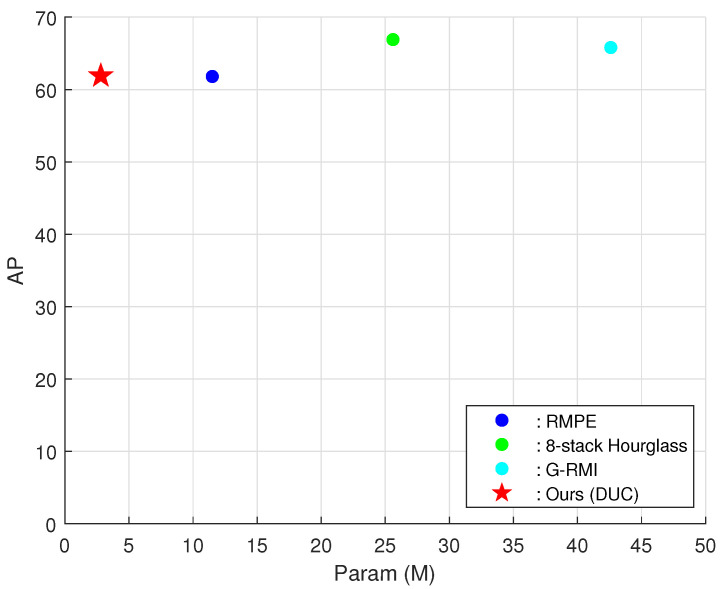
Parameter and accuracy comparison of top-down pose networks.

To further clarify the effectiveness of our scheme, we conducted additional experiments and modified only for the top-down algorithms using the same approach as the proposed method and fairly and accurately compared the amount of parameters. Table 5 below illustrates the validation results comparison of AP values, total parameters used, and FLOPS values. Our proposed model exhibits similar performance as the existing top-down-approach-based pose estimation networks and requires very few parameters in comparison as shown in Figure 6. We achieved an AP of 61.9 with only 2.80 M parameters and 1.49 FLOPS. Particularly, the amount of parameter used can be reduced by 90% compared to G-RMI with significantly lower computational complexity.

We further conducted experiments on the MPII dataset [58] to demonstrate the generalization of our model. The MPII dataset is a popular open dataset on human pose that contains 25 k images with over 40 k people with annotated pose points acquired from YouTube. We conducted knowledge distillation learning on the same teacher network and validated the performance for 16 keypoints that are different from the MSCOCO dataset. Table 6 describes the performance results of the experiment conducted on the MPII dataset. As illustrated in the table, the proposed method affords a mean of 86.9 (mean@0.5) via PCKh evaluation. We achieved a PCKh of 86.9 with 2.80 M parameters and 1.48 FLOPS. Compared to DU-Net (8), our model performed better 2.2 PCKh, and memory size decreased further to 64.6%, respectively. Moreover, in comparison with a state-of-the-art algorithm, our model achieved a balance between complexity and accuracy. Thus, the proposed pose network affords better performance than other methods.

**Figure 7:**
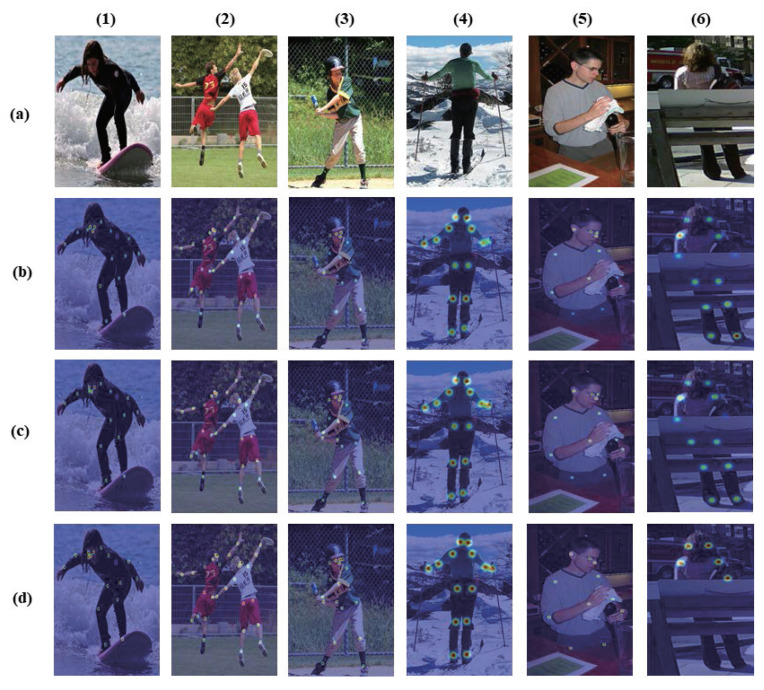
Pose estimation results of our model on the MSCOCO validation set. Column (**a**): The input images. Column (**b**): the keypoint heatmaps of Propoesd algorithm. Column (**c**): the keypoint heatmaps by the teacher model. Column (**d**): the ground-truth keypoint heatmaps. Row (1,2): Moving sequence on MSCOCO dataset. Row (3,4): Standing sequence on the MSCOCO dataset. Row (5,6): Body sequence hidden by objects in the MSCOCO dataset.

**Figure 8:**
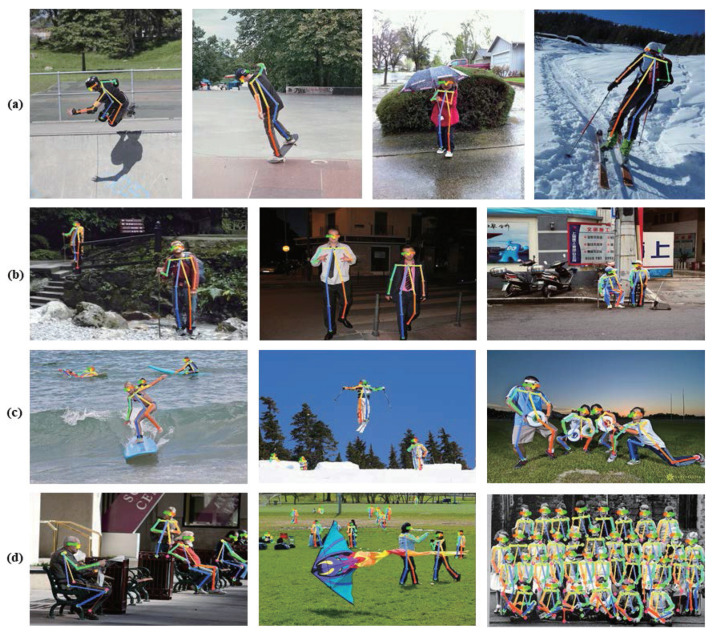
Qualitative results of our model in MSCOCO. Column (**a**): Images that contain single person. Column (**b**): Images that contain two people. Column (**c**): Images that contain three & four people. Column (**d**): Images of group of people.

Figure 7 illustrates the effectiveness of applying knowledge distillation to our method. Figure 7b–d display the pose heat map visualizations of the input image (a). (1) to (4) of Figure 7, the proposed algorithm is close to the ground-truth according to the teaching network for the moving and standing sequences. (6) of Figure 7 shows the example results when a person is occluded by an object. In fact, the ground-truth heatmap has missing keypoint labels due to occlusion object, but the teacher model identifies the missing ground-truth keypoint labels. Accordingly, the teacher model labels extra poses that assists the student network to learn as shown in 5, 6 (b) of Figure 7. Figure 8 visualizes the example image results from the validation set of the MSCOCO dataset. Therefore, our method achieved robust and stabilized pose estimation, even for difficult cases when joints are occluded by objects.

#### 4.4.2. Discussions

We focused on introducing an approach to address the imbalance between performance and computational complexity, which is a fundamental problem of pose estimation. We introduced a method that reduces complexity using a fast lightweight network with few parameters and that compensates for the insufficient performance using the knowledge distillation method. Our proposed approach does not require the designing of a new network to ensure performance or speed. Furthermore, our approach is not limited to the network capacity of the network, as well as it advantageously complements performance by combining networks in various ways.

The proposed model has a limitation: its performance is relatively lower than that of existing deep and heavy networks such as [17,18,19,20,21,22,23]. However, our lightweighting scheme has demonstrated that its performance is high even when the resources are limited, such as low memory and computing power. Furthermore, a highly efficient learning method using the DUC layer is expected for pose estimation in mobile devices or embedded devices requiring low memory size with real time.

### 4.5. Conclusions

In this paper, we propose a new lightweight top-down multi-person pose estimation approach. The main challenge of a top-down approach is the achievement of a balance between the complexity and accuracy. Traditional top-down pose estimation approaches afford high performances, but the high complexity and high computing load make them time consuming. To resolve this dilemma, we introduced KDLPN, which operates efficiently and has low computational complexity. Moreover, the pixel shuffling operation in the decoder allows the reduction of the number of parameters. We applied the knowledge distillation method to prevent performance degradation and improve accuracy. Overall, our proposed algorithm achieved a balance between complexity and accuracy, as demonstrated by the qualitative and quantitative evaluation on the MSCOCO and MPII datasets.

## Figures and Tables

**Table 1:** Decoder architecture.

Stage		Layer	Output Shape
Input			12×8×704
DUC Stage 0	PixelShuffle	PixelShuffle	24×16×176
	Convolutional Block	conv2d 3×3	24×16×352
		BatchNorm2d	
		ReLU	
DUC Stage 1	PixelShuffle	PixelShuffle	48×32×88
	Convolutional Block	conv2d 3×3	48×32×176
		BatchNorm2d	
		ReLU	
DUC Stage 2	PixelShuffle	PixelShuffle	96×64×44
	Convolutional layer	conv2d 3×3	96×64×17

**Table 2:** Results for the MSCOCO validation sets in a lightweight network with $\alpha_{KD}=0.8$.

Encoder	AP	${\mathrm{AP}}^{50}$	${\mathrm{AP}}^{75}$	${\mathrm{AP}}^{M}$	${\mathrm{AP}}^{L}$	Param (M)	FLOPS (G)
Hourglass (4-stack)	64.8	82.1	71.3	60.6	71.6	26.0	46.6
Hourglass (2-stack)	62.6	81.1	69.0	58.2	69.4	13.5	23.3
Hourglass (1-stack)	55.4	78.8	60.9	51.0	62.4	7.17	11.7
ShufflenetV2 [54]	52.5	76.9	57.5	48.2	59.1	2.73	1.26
MobileNetV3 [40]	60.8	81.1	67.9	56.2	68.0	3.94	1.36
MobileNetV2 [39]	56.1	79.0	62.0	52.1	63.0	4.54	2.12
MobileNetV1 [38]	54.8	77.9	59.9	50.1	61.7	4.69	2.11
MnasNet [55]	57.7	79.4	63.8	53.9	64.5	5.42	2.14
PeleeNet	61.9	82.0	68.5	57.6	68.7	2.80	1.49

**Table 3:** Comparison of the network parameters with $\alpha_{KD}=0.8$.

Encoder	Decoder	Decoder Param (M)	Decoder FLOPS (G)	AP
PeleeNet	Deconv (512 512 512)	14.11	34.49	62.8
	Deconv (256 256 256)	4.98	9.19	63.5
	Deconv (128 128 128)	1.98	2.58	62.8
	Deconv (64 64 64)	0.86	0.79	43.3
	Half-step deconv	5.21	4.58	63.4
	**Ours**	0.71	0.47	61.9

**Table 4:** Comparison of experiments on knowledge distillation.

Encoder	Decoder	$\alpha_{KD}$	AP
PeleeNet	DUC	0.3	59.6
		0.4	59.6
		0.5	60.4
		0.6	60.6
		0.7	61.5
		0.8	61.9
		0.9	61.6
		1.0	60.9

**Table 5:** Validation results comparison of AP values, total parameters used, and FLOPS values on MSCOCO dataset Params and FLOPS are calculated for the pose estimation network, and those for human detection and keypoint grouping are not included.

Method	Encoder	Decoder	AP	Param (M)	FLOPS (G)
RMPE	4-stack hourglass	Deconv	62.3	14.8	-
8-Stage Hourglass	Hourglass	(dev)	66.9	25.6	26.2
G-RMI	ResNet-101	(dev)	65.8	42.6	57.0
**Ours**	PeleeNet	DUC	61.9	2.80	1.49

**Table 6:** Comparison results on the MPII validation dataset (PCKh@0.5).

Method	PCKh(@0.5)	Param (M)	FLOPS (G)
DU-Net (8) [59]	84.7	7.90	-
Tang et al. [60]	87.5	15.5	33.6
Yang et al. [61]	88.5	7.30	16.2
Bulat et al. [61]	89.5	8.50	9.9
EfficientPoseIV [62]	89.75	6.56	72.9
**Ours**	86.9	2.8	1.48

## Data Availability

Not applicable.
